# Pilot examination of stress, heart rate variability, and alcohol craving and use among female veterans

**DOI:** 10.3389/fpsyt.2022.886801

**Published:** 2022-09-09

**Authors:** Cathryn Glanton Holzhauer, Elizabeth E. Epstein, Laurel Bickar, Robyn A. Ellis, Nnamdi Pole, Mehmet Sofuoglu, David A. Smelson, Kristin Mattocks

**Affiliations:** ^1^VA Central Western Massachusetts, Division of Research and Education, Leeds, MA, United States; ^2^Department of Psychiatry, University of Massachusetts Medical School, Worcester, MA, United States; ^3^Department of Psychiatry, Harvard Medical School, Boston, MA, United States; ^4^Division of Depression and Anxiety, McLean Hospital, Belmont, MA, United States; ^5^Department of Psychology, Smith College, Northampton, MA, United States; ^6^Department of Psychiatry, VA Connecticut Healthcare System, West Haven, CT, United States; ^7^Department of Psychiatry, Yale University School of Medicine, West Haven, CT, United States

**Keywords:** heart rate variability, alcohol use, craving, female, veteran

## Abstract

Rates of alcohol use disorder (AUD) are increasing among civilian and veteran populations of women in the United States, and stress pathophysiology (i.e., abnormal acute and long-term change in physiological responses to stress) is central to the maintenance of alcohol misuse within this population. Heart rate variability (HRV) is one measure of stress regulation that may help to explain the association of stress with alcohol misuse among women. In the current analysis of pilot data, 20 women veterans attended an in-person laboratory session and completed 35 daily assessments of their alcohol use and craving. During the lab session, the effects of a stress induction procedure on self-reported alcohol craving and HRV were assessed. HRV was continuously measured and indexed in the time domain, using the root mean square of successive differences between normal heartbeats (RMSSD). Alcohol craving and use during the longitudinal 35-day study period were measured *via* self-report questionnaires sent to participants' phones. Results indicated that resting HRV in the lab was positively associated with odds of daily craving. Moreover, HRV during the stressor, as measured in lab, was positively associated with (1) overall alcohol craving in the lab (i.e., with resting and post-stress craving), and (2) number of daily drinks during the 35-day study period. This pilot study suggests the potential role of HRV in response to stressors in predicting alcohol craving and use among female veterans. It provides pilot data for research on stress-reactive HRV as a biomarker for alcohol misuse among women, and discusses directions for future research.

## Introduction

Between 2000 and 2016, there was a substantial increase in alcohol use and binge drinking among civilian and veteran populations of women in the United States (1), a trend that continued up to and during the COVID-19 pandemic in 2020 (2). Lifetime Alcohol Use Disorders (AUD) among women veterans are estimated at 27% (3), with up to 32% reporting binge drinking (4). Women veterans are at greater risk of chronic and acute stress exposure (5) and alcohol misuse (3) compared to civilian women. Research on the roles of stress and negative affect in alcohol use and relapse demonstrates greater salience of these processes in women's alcohol misuse than men's (6). Women with AUD have higher rates of all mood and anxiety disorders (7, 8) and greater likelihood of drinking in response to stress and negative affect compared to men (9, 10). Furthermore, alcohol-induced alterations in emotional and biophysiological markers of adaptive stress response are more common in female participants with heavy drinking and/or AUD than male participants (6). Given these findings, there is a need for more research on mechanisms that explain the associations of stress and alcohol use among women. This study considers the role of stress regulation as indexed by heart rate variability (HRV) in explaining stress-related alcohol craving and use among female veterans.

### Heart rate variability (HRV) and alcohol use

Heart rate variability (HRV) is a measure of changes in the timing between consecutive heart beats in response to a variety of internal and external demands or to maintain homeostasis. These changes can be driven by sympathetic, parasympathetic, and other influences (11). People vary in their “resting” or “tonic” HRV levels. Among healthy individuals, higher resting HRV is viewed as a trait-like marker of individual differences in the capacity to respond effectively to situational challenges, including psychological stressors (12–14). Some scholars are also interested in studying “phasic” HRV, or changes in HRV in response to a specific stressor or other (e.g., alcohol) cue. In healthy samples, HRV tends to decrease in the immediate aftermath of a stressor. However, some studies have observed phasic increases in HRV that appear to reflect efforts to regulate the stress response (15), regardless of whether that effort is adaptive (e.g., cognitive reappraisal) or maladaptive (e.g., emotional suppression) (16). In the current paper, we will use the following terms: “resting” HRV to refer to baseline/quiet sitting and “stress-reactive” HRV to refer to the phasic change in HRV from a resting state to an induced stressed state.

Resting and stress-reactive HRV have been studied among individuals with heavy drinking and AUD. One meta-analysis of 15 studies found that patients with AUD had significantly lower resting HRV than healthy controls (*g* = −0.43, *p* = 0.01) and that this difference seemed to be mostly mediated by HRV measures with strong parasympathetic nervous system influence (17). In terms of phasic changes in HRV, pronounced restriction in HRV after an emotion or stress induction is typically associated with psychopathology (18). However, elevated HRV in reaction to emotional or stressful stimuli may reflect irregularities in parasympathetic responding, in which a more robust response occurs (19). Research on emotion regulation and HRV has found increased HRV when participants are instructed to use emotion regulation strategies (either adaptive and maladaptive strategies) to cope with an emotional or stressful situation (16, 20). Among a sample of individuals recovering from AUD, those with better self-control over alcohol craving had increased HRV in response to a drinking task, compared to those with poorer self-control over cravings (14). These findings are consistent with theory that increased phasic HRV reflects engagement of self-regulatory efforts (15).

Regarding HRV response to an appetitive stimulus (e.g., an alcohol or other substance-related cue), self-regulatory efforts in responding to the appetitive cue may similarly be associated with increased HRV (21). In the substance use literature, results on the directionality of association between phasic HRV and substance related behavior, in response to stress or not, are mixed. Results vary based on cue type (e.g., stress vs. alcohol cues), HRV measure used, and whether the participants are actively using substances or have varying times of abstinence. However, studies have found increased HRV in response to alcohol-related stimuli or stress to be associated with negative outcomes. For example, among recently abstinent participants with AUD, phasic HRV was found to increase upon exposure to negatively valanced emotion pictures cues compared to control/healthy participants, and this higher HRV after the cues was associated with higher craving (22). Another study found that patients with AUD who had higher increases in HRV in response to stress-primed alcohol cues had a greater likelihood of relapse at a 6-month follow-up, compared to patients with lower HRV cue-reactivity to stress-primed alcohol cues (23). Higher HRV in response to stress and appetitive stimuli, such as alcohol cues, may reflect greater regulatory effort required to cope with reactions. Continued research on the association of HRV with self-regulatory behaviors, including the association of resting and stress-modulated HRV with alcohol use over a longitudinal data collection period, is needed.

In sum, research suggests that individuals with heavy drinking or AUD demonstrate differential HRV responses to stress compared to healthy control participants, and this stress response is associated with alcohol craving and use. Stress-reactive, phasic changes in HRV may impact momentary and daily alcohol craving and use among women. Given that women have historically been under-represented in AUD research (24), that HRV has been identified as a potential biomarker of addiction and risk for relapse (18, 25), and that stress is closely related to alcohol misuse and relapse among women (6), these questions have important research and clinical implications. Furthermore, given the heightened chronic stress and trauma exposure among women veterans (5), stress responding n may be especially salient for understanding this population's risk for AUD and alcohol misuse more generally.

### Aims and hypotheses

The first study aim was to test the associations of stress-reactive, phasic HRV with stress-reactive, phasic alcohol craving among female veterans in the lab. It was hypothesized there would be positive associations of stress-reactive HRV and craving, given research cited above showing that, although phasic HRV is inversely associated with emotional responding, this association may differ among individuals with AUD and in association with alcohol craving. The second aim was to examine the association of stress-reactive HRV in the lab to longitudinal measures of alcohol craving and use across a 35-day period. This aim examines whether the HRV assessed in the lab in aim 1 are also associated with craving and drinking during daily life.

## Methods

### Participants

Data for these pilot analyses were drawn from a larger, on-going clinical trial. In that study, half of the participants are taught an emotion regulation strategy (cognitive reappraisal, compared to a psychoeducation control group) during a single 50-min session. This single intervention focuses on teaching participants in that condition how to down-regulate negative affect and does not address alcohol use or cravings or encourage or teach skills for participants to change their drinking. The intervention was delivered at the start of the 35-day study period. Preliminary analyses revealed no condition-related effects on craving or drinking in this pilot sample and there was a lack of power to demonstrate such an effect of condition with the current pilot sample. Participants were 20 women veterans recruited from US Veteran Affairs Medical Centers in New England. Recruitment was conducted *via* flyers, provider referrals, and medical record reviews with letters sent to potentially eligible participants. Inclusion criteria were: (1) age 18 and older; (2) current unhealthy alcohol use, defined as scoring 3 or higher on the Alcohol Use Disorder Identification Test, Concise [AUDIT-C; see below for measure details; note: women veterans with AUDIT-C scores of 3+ have been found to have increased rates of alcohol-related consequences and blackouts, tolerance, and self-reported need to cut down on use (26)]; the average full AUDIT score for this sample was 8 (*SD* = 4); (3) if using other illicit substances, alcohol is their primary substance of use; (4) alcohol use in the past 45 days; (5) able to write and speak in English; (6) served in the U.S. military; (7) willing to provide blood samples at laboratory sessions to assay hormone levels and take urine ovulation tests at home. All inclusion criteria were established *via* participant self-report during the phone screen and confirmed at session 1. Exclusion criteria were: (1) psychotic symptoms or uncontrolled bipolar disorder; (2) brain damage or were in an accident that affects ability to complete the computerized task; (3) current (past 3 months) active suicidal ideation or intent; (4) current pregnancy; (5) currently receiving treatment for alcohol use.

### Procedures

Procedures were approved by the Institutional Review Board. Participants attended a total of 5 sessions, with data for current analyses being collected in the first 3 of those sessions. Participants also completed 35 days of questionnaires, with the start of the 35 days coinciding with their session 2. After an initial phone screen, session 1 (intake session) including study consent, baseline self-report measures, and a clinical intake.

In session 2, study staff used a manualized procedure (27) to develop a personal stress script that was used as a stress induction in the experimental session. That procedure required participants to verbally describe an event which they found “most stressful,” as staff recorded the details provided. This event was one that the participant rated as an 8 or higher on a 1–10 scale, where 10 was defined as the most stressful thing that they experienced in the past year; however, participants were encouraged to use an event that is “common in today's world, such as an argument with a loved one.” Events that were traumatic were avoided to maintain consistency between participants who did and did not have posttraumatic stress disorder, as well as to avoid a trauma response as opposed to a non-traumatic stress response. Events concerning substance use were also avoided, so as not to directly influence alcohol cravings which were evaluated before and after each stress induction. If participants provided a trauma or substance-related event, the interviewer re-directed them and reminded them to choose a stressful event that is a common event and/or did not occur in the context of substance use. Participants were asked to briefly describe the event in mind, and study staff guided them to an appropriate stressful event. The participants were asked to describe the event in detail with special attention given to their thoughts and actions. The staff then followed manualized procedures to generate the personalized stress induction scripts during the experimental (third) session.

In relation to a parent study aim that is focused on ovarian hormones, and based in hypotheses that these hormones influence stress reactivity among female participants, each participant with regular menses was then urn randomized to start their 35-day study period and have their experimental laboratory session in either the early follicular phase or mid-luteal phase of their menstrual cycle. Women who did not menstruate (i.e., due to medications, medical conditions, or menopause) were scheduled independent of any menstrual cycle status. This ensured that, for women who do experience expected hormonal fluctuations and elevated levels of progesterone (the hormones expected to be associated with HRV and stress response), those levels would be randomly distributed at the time of the experimental session.

Across the 35-day period, participants completed daily questionnaires *via* REDCap (Research Electronic Data Capture) electronic data capture tools (28, 29). REDCap is a secure, web-based software platform designed to support data capture for research studies. Questionnaire links were sent to participants' phone *via* text at 9 am each day. Participants provided information about their alcohol consumption and cravings pertaining to the past 24 h.

In the experimental session, a continuous recording of each participant's electrocardiogram (ECG) was made following procedures that are described in greater detail in the Measures section below. Participants completed outcome measures of alcohol craving and affect after a 5-min (“resting”) baseline period, during which they were asked to sit quietly. They then completed a stress induction (3.5 min) focused on their selected stressful scenario. HRV during this stress induction is used as the independent variable in analyses (“HRV during the stressor”; when controlling for resting HRV in both statistical models, this represents phasic “stress-reactive HRV” in association with alcohol craving and use). Participants were instructed to close their eyes and imagine that the situation was “happening right now.” They were told to “become completely involved in the situation, by involving your mind and body and actually doing what is being described.” After the stress induction, they once again completed the measures of alcohol craving and affect (“post-stress”). At the end of study participation, participants were compensated up to $300 gift cards for their time, the exact amount based on their session attendance and completion of daily questionnaires.

### Measures

#### Clinical interview at session 1

##### Structured clinical interview for the diagnostic and statistical manual−5 (SCID-5)

During session 1, all participants completed the SCID-5 (30) with a study assessor. Specifically, all participants were interviewed using modules that assess mood disorders, psychotic disorders, substance use disorders, and posttraumatic stress disorder.

##### Timeline follow-back (TLFB)

The TLFB (31) uses a calendar and other memory aids to determine an individual's drinking over a specified time. At baseline, participants were interviewed about their alcohol use on each of the 45 days prior session. The TLFB has excellent reliability and validity for alcohol use (32). TLFB data were used to calculate the baseline percentage of days drinking (PDD).

#### Self-report measures, session 1 and experimental session

##### Alcohol use disorders identification test (AUDIT)

The 10-item AUDIT (33) assesses alcohol consumption, drinking behaviors, and alcohol-related problems. Items are scored on a scale of 0–4, for a total possible score of 0–40. A total of 8 or more indicates hazardous or harmful alcohol use. The AUDIT-C (concise) was assessed at phone screen, and full AUDIT was administered at intake session 1.

##### Alcohol craving questionnaire short-form revised (ACQ-SF R)

The 12-item ACQ-SF (34) assesses alcohol cravings in the current moment (e.g., “If I used alcohol, I would feel less tense”). Each item is scored on a 7-point scale from “strongly disagree” to “strongly agree,” and items are averaged to generate a total score. Two ACQ total scores were used in analyses: (1) at baseline/the start of experimental session (“resting” or tonic ACQ), and (2) after the stress induction (“post-stress” ACQ). These 2 administration time points allows for the examination of change in craving in response to stress.

##### Positive affect negative affect schedule (PANAS)

The 20-item PANAS (35) assesses positive and negative affect in the moment. Each emotion (e.g., enthusiastic, irritable, nervous) is rated on a 5-point scale from “very slightly or not at all” to “extremely.” Answers are summed to create positive affect and negative affect scores, each comprising 10 items and ranging from 10 to 50. The PANAS was administered at baseline and after the stress induction during the experimental session, as outlined above for the ACQ.

#### Biological measures, experimental sessions

##### Heart rate variability (HRV)

After appropriate skin preparation, three BIOPAC EL503 series disposable electrocardiogram (ECG) electrodes were attached below the left and right clavicles and the reference electrode was placed on the lower left side of the ribs. Lead 100 series leads were attached to these electrodes and taped down to minimize movement artifact. Participants were seated and instructed to keep movement to a minimum while ECG data were collected. A BIOPAC MP160 data acquisition system (Biopac Systems, Inc., Goleta, CA, USA), with an ECG100C amplifier, continuously sampled ECG at 1,000 Hz. The raw ECG waveforms were visualized and analyzed using BIOPAC *AcqKnowledge* software version 5.05. Waveforms were visually inspected for artifact prior to data collection, to ensure proper electrode placement, and then examined again offline afterwards. Event markers were added to the file to indicate the timeframes during the resting baseline and during the stress induction, which were the only heart beats included in the HRV analyses. No significant artifact was observed in these time windows. Among possible HRV measures, we selected the root mean square of successive (inter-beat interval) differences (RMSSD), a time-domain measure that is calculated by determining and squaring the difference between each heartbeat in milliseconds, which are then averaged together before the square root is applied. RMSSD is one of the most widely reported measures of HRV because of its good reliability over relatively short durations (such as those used in this study) and because it is considered the primary time-domain measure to estimate parasympathetic influences on cardiac activity in a way that is relatively unaffected by breathing artifact (11).

#### Daily alcohol use and craving questionnaires

*Via* REDCap, as described above, participants were prompted *via* text over the course of 35 days to provide information about their alcohol use and alcohol craving over the past 24 h. Specifically, participants were asked “Did you have cravings for alcohol in the past 24 h?” and “Did you drink alcohol in the past 24 h?” If participants responded “yes,” follow-up questions about frequency and intensity were asked. Exact number of drinks were collected *via* TLFB on a weekly basis. For the current analyses, we used dichotomous yes/no to indicate whether participants reported alcohol cravings over the prior 24 h and used the reported number of drinks per day as the outcome variables for aim 2.

### Data analysis plan

Distributions of the variables of interest were examined and independent variables were grand mean-centered prior to running analyses. Bivariate correlations of baseline AUDIT score, HRV during the stress induction, and subjective negative affect and alcohol craving at baseline and after the stress induction (“post-stress”) were calculated.

Aim 1 was to examine the association of stress-reactive HRV (i.e., resting RMSSD as a covariate, with RMSSD during the stressor as the independent variable) as the independent variable with stress-reactive alcohol craving (i.e., baseline craving as a covariate, with post-stress craving as the dependent variable) among female veterans in the lab. A repeated measure general linear model was conducted. Menstrual cycle status [i.e., women with regular cycles (*n*= 11) compared to without regular cycles (*n*= 7)], post-stress negative affect, and resting levels of RMSSD were entered as covariates, and RMSSD during the stressor was entered as the main independent variable of interest. Resting RMSSD was added as a covariate to statistically model phasic change in HRV. Post-stress negative affect was included as a covariate, given the well-established association of negative affect with craving and expected individual variability in intensity of the stress response to the induction. Baseline alcohol craving and post-stress alcohol craving in the lab were entered as the two measures of the repeated outcome. Within- (change between the two timepoints of craving—baseline and post-stress) and between- (collapsed across time) subject effects of our predictors on craving were examined. Aim 1 analyses were conducted utilizing Statistical Package for the Social Sciences (SPSS) Version 22.

Aim 2 was to again examine the association of stress-reactive HRV with alcohol craving, but using craving data from participants' 35 daily assessment logs as the dependent variable in the first model and number of drinks per day as the dependent variable in the second model. Two mixed level models (MLMs) were run using R software, one for daily report of alcohol craving (yes/no, whether craving was experienced that day) and one for daily number of standard drinks of alcohol. For the first model, predicting daily craving, the *glmer* package was used with model fit by maximum likelihood and Laplace approximation, and binomial fit given the dichotomous outcome. These models provide the estimated probability of craving on a given day, based on the predictors included in the model. The second model, testing the HRV-daily drinks association, used the *lmer* package and a linear mixed model was fit by restricted maximum likelihood (REML), with *t*-tests for significance using Satterthwaite's method. Both models included as predictors the intercept, menstrual cycle status (having regular menses or not, as in aim 1), resting RMSSD, then RMSSD during the stressor. All continuous variables were grand-mean centered, as such the intercept can be interpreted as adjusting for all included predictors at an average point in the 35-day study period. First, null models were fit to examine variation in the outcome (i.e., cravings, drinks) across individuals. Next, models were fitted in a forward stepping procedure, adding each predictor of interest with relevant covariates into the model one at a time. To illustrate, the full equation for cravings is as follows:


$$\begin{array}{l} \eta_{ti}=\beta_{00}+\beta_{01}\left(menstrual\;cycle\;status\right)\\ \;+\beta_{02}\left(HRV_{resting}\right)+\beta_{03}\left(HRV_{\;stress}\right)+r_{0i} \end{array}$$

In this model, η_*ti*_= log($\frac{\emptyset_{ti}}{{1-\emptyset }_{ti}}$) reflects the log-odds of reporting cravings, *β*_00_= the average log-odds of reporting cravings across individuals, and *β*_01_ through *β*_03_ reflect the effects of the covariates and HRV during the stress induction (“HRV stress”) on the log-odds of reporting cravings. An identical, albeit linear, model was built as illustrated for alcohol drinks per day as the outcome variable.

## Results

### Descriptive statistics

See Table 1 for descriptive statistics and Table 2 for Pearson correlation coefficients. Significant, positive correlations were found for HRV during the stressor with baseline (*p* = 0.02) and post-stress alcohol craving (*p* = 0.04). Near-significant positive associations were found for resting HRV with post-stress craving (*p* = 0.07) and HRV during the stressor with AUDIT total score (*p* = 0.055).

**Table 1:** Participant demographics and descriptive statistics (*n* = 20).

	**M/N**	**(SD)/%**
**Age (years)**	44	(13)
**Education (years)**	17	(2)
**Annual household income**	$78,922	($57,756)
**Percent days drinking (45 days prior to session 1)**	60%	(31%)
**Total baseline AUDIT score**	8	4
**Lifetime AUD diagnosis**	12	60%
**Race**	—	—
White	18	90%
Mixed race	1	5%
Asian	1	5%
Hispanic ethnicity	1	5%
**Sexual orientation**	—	—
Heterosexual/straight	17	85%
Bisexual	3	15%
**Employment**	—	—
Full-time	13	65%
Part-time	2	10%
Student, retired, disability, homemaker	4	20%
Unemployed	1	5%
**Marital status**	—	—
Single	8	40%
Married	3	15%
Living as married	1	5%
Separated	1	5%
Committed relationship	5	25%
Widowed	2	10%

**Table 2:** Correlation (Pearson's *r*) of variables of interest.

	**RMSSD**		**ACQ**		**PANAS negative affect**	
	**Resting**	**During stressor**	**Resting**	**Post-stress**	**Resting**	**Post-stress**
ACQ	Resting	0.37	0.54*	—	—	—	—
	Post-stress	0.43^†^	0.49*	—	—	—	—
PANAS negative affect	Resting	0.12	0.16	0.25	0.12	—	—
	Post-stress	0.06	−0.01	0.27	0.24	—	—
Baseline AUDIT	0.14	0.46^†^	0.72**	0.71**	0.29	0.25

^**^Correlation is significant at 0.01.

*Correlation is significant at 0.05.

^†^Correlation *p* <0.1 (2-tailed).

ACQ, Alcohol Craving Questionnaire (total score); PANAS, Positive Affect Negative Affect Scale; AUDIT, Alcohol Use Disorder Identification Test.

“Resting” refers to baseline, while participant sits quietly, “During Stressor” refers to the time frame during which participants listen to the stress induction script, and “Post-Stress” refers to ratings after a personalized stress induction procedure. Correlations reflect associations among the full sample, including women without regular menstrual cycles.

### Aim 1

Repeated measure general linear models tested HRV as statistical predictor of alcohol craving, as measured in the lab. Multivariate tests showed a non-significant association of time-by-RMSSD during the stressor with alcohol craving at baseline and post-stress, *F* = 0.09, *p* = 0.77. The time-by-resting RMSSD was also non-significant, *F* = 2.48*, p* = 0.14. Tests of between-subject effects, however, showed a significant association of RMSSD during the stressor with alcohol craving across time points, *F* = 7.15, *p* = 0.02, Cohen's *f* = 0.64, such that higher HRV during the stress induction was associated with higher overall alcohol craving reported in the lab. The between-subjects association of resting RMSSD with alcohol craving across timepoints was non-significant, *F* = 2.41, *p* = 0.15, Cohen's *f* = 0.31. Other covariates were non-significant, all *p* < 0.05.

### Aim 2

Aim 2 examined the association of HRV, measured in the lab, with daily alcohol craving and alcohol use across the 35-day study period. For both aim 2 models, the null models showed that the random effects varied significantly (*p*s < 0.05). Two metrics of clustering in the data were calculated from the null models, specifically the interclass correlation coefficient (ICC) and design effects (DEFF). Greater values on these metrics indicate greater impact of clustering on the data, with ICC values about 0.20 and DEFFs >2 suggesting clustering should not be ignored in the data. For both outcomes, ICC and DEFFS were above recommended cut offs (ICC = 0.83, 0.55; DEFF = 25.38, 17.32 for craving and drinks models, respectively), suggesting that MLM is an appropriate statistical approach. Further, multicollinearity was explored for predictors in the models; variance inflation factors ranged from 1.18 to 1.35, suggesting that there was not significant multicollinearity between the predictors included in the models.

See Table 3 for results of aim 2 models. The model predicting daily alcohol cravings showed that for every unit increase in resting HRV there was a 0.07 increase in the log-odds of reporting craving on daily assessments (*z* = 1.96, *p* = 0.049, OR = 1.07). HRV during the stressor was not significantly associated with odds of reporting craving on daily logs (*p* = 0.40). The model predicting daily number of drinks consumed indicated the opposite, with HRV during the stressor significantly associated with number of drinks consumed (B = 0.015, *t* = 2.38, *p* = 0.032). Again, higher HRV during a stress induction in the lab was associated with more drinks during the study period. Resting HRV (RMSSD; *p* = 0.91) and status of menstrual cycle (*p* = 0.49) were not associated with the number of drinks during the study phase.

**Table 3:** Aim 2 model results.

	**Cravings (binomial)**					**Number of drinks (linear)**				
**Predictors**	**Estimates**	**SE**	***z***	***p***	**OR**	**Estimate**	**SE**	**df**	***t***	***p***
(Intercept)	−3.04	1.27	−2.39	0.017	-	1.55	0.47	14	3.27	>0.001
Cycle	0.50	2.08	0.24	0.811	1.65	0.59	0.83	14.07	0.71	0.492
Resting RMSSD	0.07	0.04	1.96	0.049	1.07	−0.001	0.013	14.11	−0.11	0.914
RMSSD during stressor	0.01	0.02	0.83	0.405	1.01	0.01	0.01	14.06	2.38	0.032

Cycle is coded 1 = regular menstrual cycle, 0 = without regular cycle.

OR, Odds ratio.

## Discussion

The current study examined the associations of heart rate variability (HRV) in response to a personalized stress induction with alcohol craving and use among a sample of female veterans. A growing body of research suggests the important role of psychological and physiological stress reactivity in alcohol use disorder (AUD) among women, and women veterans are at heightened risk of chronic stress and trauma exposure. In the current study, higher stress-reactive HRV (i.e., HRV during the stressor, while controlling for resting HRV) was associated with higher alcohol craving and use among female veterans, in the lab and in daily life. In the lab, higher HRV in response to the stress induction was associated with higher overall craving (i.e., craving reported at baseline and after the stressor). When examined in relation to “real life” alcohol use, this higher HRV as measured in the lab was associated more drinks per day. While these pilot data indicate the important role of stress-reactive HRV in alcohol use among women—in the lab and in “real life”—the results need to be replicated with the larger sample. Although not the focus of these pilot analyses, results also demonstrated a positive association of *resting* HRV with odds of daily craving. The effect of these findings, however, was small, particularly in comparison to the findings regarding HRV during the stressor with craving and use. Collectively, however, these results suggest the relevance of HRV as a factor in women's alcohol use and highlight changes in HRV in response to stressful life events as a potential biomarker of the intensity of women's drinking, even among non-treatment seeking individuals.

The results demonstrating a positive association of stress-reactive HRV with alcohol craving are consistent with previous research (22, 23); this study extends that work to demonstrate the associations of HRV during the stressor with drinking over the 35 study days, within a sample of female veterans. As suggested in the introduction, such findings initially seem contradictory to general findings on HRV and psychopathology, which broadly support an association of higher HRV with better physical and mental health states. However, they are supportive of findings and hypotheses, outlined above, that increased HRV in this population may reflect heightened efforts at self-regulation (whether or not those efforts are adaptive). It is feasible that the positive association of HRV with craving in the lab, in this sample, reflects increased stress or emotion regulatory efforts, and/or attempts at down-regulating, or suppressing, cravings in response to stress. It is notable, however, that this was not a treatment seeking sample and therefore the participants may not have been actively regulating alcohol cravings. In this case, increased HRV may be more reflective of increased stress (not necessarily craving) regulation efforts. Alternatively, increased craving in response to stress may reflect participant's habitual use of alcohol as a maladaptive regulation strategy itself (i.e., participants have learned that alcohol use is a strategy used for reducing stress or negative emotion and craving reflects a regulatory effort in the moment). In this case, increased HRV correlated with increased craving and drinking may reflect a classically conditioned response to stress in which alcohol follows stress and directly impacts HRV among regular alcohol users (23). These, along with the other findings, require continued research among women. In particular, research that experimentally manipulates emotion and craving regulation in this population can help elucidate these findings.

### Limitations

A primary limitation of this pilot study is its small sample size. The findings are intended to inform future research questions and should be interpreted cautiously until they can be replicated in a larger sample. The small sample size also limited our statistical power to detect small effects and prevented us from conducting some statistical tests that we might otherwise have run. Concern about making Type I errors prevented the addition of other cardiac measures to our analyses (e.g., heart rate, low-frequency HRV), which may, in the future, help us to better interpret the biological mechanisms driving our findings.

### Conclusion and future directions

Data collection for the current study is ongoing. While preliminary, these findings and relatively large effect sizes warrant continued research that will allow us to address the limitations raised above. The findings from this study extend past research, using longitudinal data collection on alcohol craving and use among a non-treatment seeking population of female veterans. There is a deficit of prospective research with female veterans, who are at increased risk for stress-related disorders including AUD. Future analyses will include clinical diagnoses of posttraumatic stress disorder and lifetime trauma exposure, as these conditions—in addition to chronic alcohol use—have been shown to directly impact stress reactivity and regulation (36) and may serve to further our understanding of the stress-alcohol association in this population.

Given the focus on women, the larger study will also examine the potential moderating role of the ovarian hormone progesterone in the association of HRV with alcohol craving and use. HRV appears to vary across women's menstrual cycle in accordance with fluctuations of progesterone (37–39), and progesterone has been shown to influence women's physiological stress responses (40, 41), as well as alcohol craving and use among women with heavy alcohol use and/or AUD (42). While menstrual cycle status (has regular menstrual cycles/does not) was entered as a covariate in these analyses, examining HRV response to stress and its associations with alcohol use among those women with fluctuating hormone levels is an important next step. The potential implications of hormones for women's self-regulatory behavior and HRV responding has not been explored.

HRV predicted substantive variance in craving among this sample of women veterans, even with a small sample size, providing additional support for HRV as a proximate measure of stress and alcohol craving and use. Collectively, understanding and targeting the biological mechanisms which contribute to stress-induced alcohol use has the potential for enhancing treatments, predicting use, and providing care tailored to the needs of women.

## Data availability statement

Datasets are available in accordance with local and national guidelines regarding data sharing. Requests to access the datasets should be directed to cathryn.holzhauer@va.gov.

## Ethics statement

The studies involving human participants were reviewed and approved by VA Connecticut Healthcare System Institutional Review Board. The patients/participants provided their written informed consent to participate in this study.

## Author contributions

CH: study conceptualization, data curation, statistical analysis, funding acquisition, methodology, project administration, supervision, and writing and editing the manuscript. EE, MS, DS, and KM: assisted with study conceptualization, planning of methodology, supervision, funding acquisition, and editing the manuscript. NP assisted with planning the psychophysiological methodology and contributed to the writing about heart rate variability. LB coordinated the study, assisted with data curation, project administration, and editing of the manuscript. RE assisted with data curation, project administration, statistical analyses, and writing and editing of the manuscript. All authors contributed to the article and approved the submitted version.

## Funding

This work was supported by Department of Veterans Affairs, Veterans Health Administration CSR&D grant CX001951 (PI: CH). The opinions expressed here are those of the authors and do not represent the official policy or position of the U.S. Department of Veterans Affairs or the U.S. Government.

## Conflict of interest

The authors declare that the research was conducted in the absence of any commercial or financial relationships that could be construed as a potential conflict of interest.

## Publisher's note

All claims expressed in this article are solely those of the authors and do not necessarily represent those of their affiliated organizations, or those of the publisher, the editors and the reviewers. Any product that may be evaluated in this article, or claim that may be made by its manufacturer, is not guaranteed or endorsed by the publisher.
